# Diet in Disease

**Published:** 1892-10-15

**Authors:** Ernest Hart

**Affiliations:** Bachelier-ès-Sciences-es-Lettres (rèstreint), formerly Student of the Faculty of Medicine of Paris, and of the London School of Medicine for Women


### DIET IN DISEASE.

By Mrs. Ernest Hart, Bachelier-ès-Sciences-es-Lettres (rèstreint), formerly Student of the Faculty of Medicine of Paris, and of the London School of Medicine for Women.

### III.—STIMULANTS.

### Alcohol.

There is no subject respecting which such opposite opinions have been expressed as on the value of alcohol as an article of diet, and there is also no subject on which it is more difficult to express an unbiassed opinion based on the clear and unmistakeable evidences of science.

**Is Alcohol a Food?**—This has been seriously denied by some scientists who have sought to prove, in support of their assertions, that all the alcohol taken could be recovered again in the breath, the perspiration, and the excretions; and hence that it played no part in tissue change. This, however, is not the fact. Only a small amount of the alcohol taken can be recovered, and the question remains; what becomes of it in the body? Dujardin-Beaumetz, the French physician and pharmacologist, who founds his opinions on practical experiments, gives the following answer to this question. He states that if alcohol is taken in small quantities, it passes into the blood, where it acts upon the red blood corpuscles, which are, as is well known, the oxygen-carriers of the body, and it obliges them to part with their oxygen. By this action, alcohol diminishes the oxidation of the tissues, and is therefore, though not a true food itself, a substance which lessens the necessity for food, thus being what he calls *un aliment d'epargne*, or as we may translate it, an economiser of food, or a tissue waste-preventer. If taken in larger quantities, too much oxygen is extracted from the blood-corpuscles and the temperature of the body falls, owing in part to deficient oxidation. If taken in still larger quantities, the whole of the alcohol does not undergo combustion, but circulating freely in the blood it acts directly on the cerebro-spinal system, producing excitement, narcotism, and the symptoms of intoxication.

**The Effects of Alcohol.**—Alcohol has the effect of stimulating the cardiac centres and of increasing the number and volume of the heart-beats, and also of dilating the arterioles, thus temporarily producing a sensation of warmth and comfort which is frequently succeeded by chilliness, caused in some measure by the greater surface of blood exposed to the influence of skin radiation. The lowering of the temperature of the body by large doses of alcohol is a well known fact, and alcohol consequently was at one time advocated and used by certain physicians to reduce the temperature in acute fevers. This method of treatment has now been discontinued, as it was found that the remedy was as bad as the disease, and that in reducing temperature by alcohol, which is less certain in its effects than other available anti-pyretics, various morbid conditions and complications were produced. The depression of temperature in the coma of intoxication is, moreover, so well-known to the persons who have to deal with these unfortunate cases that in the Glasgow lock-ups large fires are kept alight on Saturday nights, before which the dead-drunk brought in may be laid, in order that they may not perish of cold. Alcohol is one of those strange substances which have the power of producing apparently opposite results. In small quantities it stimulates the action of the heart, in large, it depresses it; in small quantities it increases the secretion of gastric juice, in large, it destroys the pepsin and arrests digestion; in small quantities it has an exhilarating effect on the nervous system, in large, it is narcotic. If, therefore, the production of the stimulating action of alcohol is required, the question which it is important to answer is, what is the amount which can be taken without exceeding the narrow limit beyond which alcohol is harmful?

**The Amount of Alcohol which can be taken with Impunity.**—There is a general consensus of opinion on the part of physicians that from 1 to 1½ oz. of pure alcohol is the maximum amount which a healthy man should take in twenty-four hours. Translated into common parlance, this means from 2 to 3 oz. of brandy or whiskey, from 4 to 6 oz. of port or sherry, from 10 to 15 oz. of champagne or burgundy, and from 1 to 1½ pints of beer or porter. More than this is harmful. Persons under forty years of age, in whom the digestive functions are normally performed, and who show no signs of nervous disturbance or degeneration, do not generally require alcohol at all, and are healthier and better and likely to live longer without it. For persons over forty, in whom digestion has become impaired by anxiety, confinement in close rooms and offices, sedentary or unhealthy occupations, or in whom nervous energy is exhausted or deficient, a small amount of alcohol in the form of wine or beer, taken with the food, is a useful stimulant. By its influence the secretion of gastric juice is increased, and digestion thereby promoted.

**The almost universal use of fermented alcoholic drinks with meals,** in all times, nations and ages, and the facility with which the fruits of the earth ferment and produce alcohol, seem to point not only to a human need, but to the supply of such a need by Nature. Alcohol is, however, like many of the other gifts of Nature, the use of it is beneficial, the abuse baneful. It is, in fact, a matter of common experience that, taken in moderate quantities, "the appetite is augmented, digestion is promoted, the nervous system stimulated, and the mental faculties exhilarated by alcohol" (Pavy). Dr. King Chambers picturesquely puts it: "That everybody recognises in alcohol a power of blunting sorrow and pain, of checking the sensation of weariness, mental and bodily, of taking the points off the stings and buffets, discomforts and nastiness of daily life; but also of corrupting the delicate appreciation of its higher delights; in short, of diminishing the sensibility to impressions in mind and body, and of lowering the receptive functions of the nervous system," and he urges that for healthy persons alcohol should never be taken as a stimulant or preparation for work; but only as a defence against the injury done by work, whether of mind or body, and that it is therefore best taken with the evening meal or after toil.

**The Pernicious Habit of Taking Nips.**—Whatever, however, may be the opinion or judgment, based on experience or science, as to the value or the reverse of taking some form of alcohol with the meals, there is no doubt that the custom of taking wine or spirits or beer between meals and on an empty stomach—in one word the pernicious habit of "nipping"—is highly injurious. The morning nip, between breakfast and the midday meal, which is so frequently taken by domestic servants, nurses, workpeople, and "City men," renders the taker less fit for his daily work than he would otherwise be, and is often in women the first fatal step towards dram-drinking, and the shameful life of the woman-drunkard, of which we are hearing so much at the present time. The flushing of the face, caused by the dilatation of the small blood vessels, usually induced by alcohol when taken alone, is symptomatic of what takes place in the stomach. The direct action of alcohol on the mucous membrane is to produce temporary congestion or blushing of the internal surface of the stomach. This congestion ultimately becomes chronic if "nips" or "drams" of spirits are frequently indulged in, with the result that the mucous membrane becomes thickened and indurated, a quantity of tenacious mucus is secreted, the digestive ferment is paralysed or destroyed, and alcoholic dyspepsia is established.

**Does Alcohol Give Strength?**—In contradiction of the popular fallacy that spirits and beer give strength and enable a man to do his work better, there may be quoted the experience of soldiers on the march in the tropics, of explorers toiling day after day across the frozen oceans of the North Pole, of Alpine climbers undergoing great fatigue. In all these instances the conclusive and invariable experience is that alcohol, taken in any form during the period of exertion, causes increased fatigue and depresses the spirits; on the other hand, in the opinion of many, a small amount of alcohol, taken after the work of the day is done, proves sedative and harmless.

**The Use of Alcohol in Disease.**—Much as opinions, however, may differ respecting the value of alcohol as an article of diet in health, or in a condition of slightly impaired health and vigour, there can be no doubt that in the hands of the physician, alcohol is one of the most valuable drugs he possesses. In the period of depression and "resolution" in acute pneumonia, in syncope, in the sudden enfeeblement of the heart from fright, accidents, or loss of blood, in ischæmia of the blood vessels of the brain, in acute fevers when the heart must be supported, in collapse, and in certain degenerative nervous and cutaneous diseases, alcohol is invaluable, and there is no drug in the pharmacopœia which can take its place. But even in these cases it must be used with caution and under medical advice, and it may be accepted as an axiom that small doses frequently repeated are always more efficacious and less dangerous than large doses at long intervals. The pulse is to the physician, and to the intelligent nurse watching beside a patient in a state of profound depression, the indication of the amount and the frequency of the dose of alcohol to be administered. I have within my own experience seen, more than once, a patient pulled through the imminently fatal exhaustion of acute pneumonia during the period of the rapid fall of temperature, by minute and oft-repeated doses of brandy, given immediately the pulse was felt to flag; and I have seen similar cases die for the want of the use of alcohol at this critical time. In the debility of convalescence, "a little wine," recommended by St. Paul to Timothy "for the stomach's sake," may promote digestion if taken just before meals; but this is again a matter for the physician to decide.

**The Evil Effects of Alcohol** when taken in excess are notorious, and I need not enlarge upon them here. It is the abuse of alcohol which fills our hospitals, our prisons, our lunatic asylums, and our workhouses, which is the fertile parent of vice and crime, the foster-mother of pauperism, and the constant generator of chronic poverty. But besides the evils which can be traced directly to dram-drinking, tippling and excess; who can gauge the number of cases in which the bread-winner dies from acute disease, from which the alcoholic habit deprives him of the power to rally; in which the children are born with degenerate constitutions; in which the will power for good is weakened, and character and happiness destroyed? It is not to be wondered at that, seeing the evils wrought by abuse, the use of alcohol is condemned, and the cry is raised, "Come out of the unclean thing, touch not, taste not, handle not." But the conventual and monastic view is not that of human life. The wise man is he who can enjoy all the good gifts of this world while keeping his body in subjection, his mind pure and his life undefiled. To acquire property is not to be a thief, to be a husband is not to be a profligate, and to drink wine is not to be a drunkard. "Be ye temperate in all things" is the dictum both of the philosopher and of the prophet.

